# Periodontal health related–inflammatory and metabolic profiles of patients with end-stage renal disease: potential strategy for predictive, preventive, and personalized medicine

**DOI:** 10.1007/s13167-021-00239-0

**Published:** 2021-04-22

**Authors:** Xiaoxin Ma, Yongli Wang, Hongyu Wu, Fei Li, Xiping Feng, Yingxin Xie, Danshu Xie, Wenji Wang, Edward Chin Man Lo, Haixia Lu

**Affiliations:** 1 Department of Preventive Dentistry, Shanghai Ninth People’s Hospital, College of Stomatology, Shanghai Jiao Tong University School of Medicine, National Clinical Research Center for Oral Diseases, Shanghai Key Laboratory of Stomatology, 639 Zhizaoju Road, Shanghai, China; 2 Laboratory of Oral Microbiota and Systemic Diseases, Shanghai Ninth People’s Hospital, College of Stomatology, Shanghai Jiao Tong University School of Medicine, National Clinical Research Center for Oral Diseases, Shanghai Key Laboratory of Stomatology, Shanghai, China; 3 Department of Nephrology, Shanghai Ninth People’s Hospital, College of Stomatology, Shanghai Jiao Tong University School of Medicine, 639 Zhizaoju Road, Shanghai, China; 4 Dental Public Health, Faculty of Dentistry, University of Hong Kong, 34 Hospital Road, Hong Kong, China

**Keywords:** Predictive preventive personalized medicine, Chronic kidney disease, End-stage renal disease, Disease-specific metabolic profile, Mass spectrometry, Gingival crevicular fluid, Interleukin-8, Interleukin-6, C-reactive protein, Serum, Periodontal health, Metabolites, Inflammation, COVID-19

## Abstract

**Objectives:**

To compare the periodontal health related–inflammatory and metabolic differences between patients with end-stage renal disease (ESRD) and healthy controls (HC), and to identify potential biomarkers in gingival crevicular fluid (GCF) and serum of ESRD patients for predictive, preventive, and personalized medicine (PPPM).

**Methods:**

Patients with ESRD (ESRD group; *n* = 52) and healthy controls (HC group; *n* = 44) were recruited. Clinical periodontal parameters were recorded. The differential metabolites in the GCF and serum were identified by liquid chromatography/mass spectrometry (LC/MS). Inflammatory markers including interleukin-1β (IL-1β), interleukin-6 (IL-6), interleukin-8 (IL-8), and C-reactive protein (CRP) were also assessed.

**Results:**

In the ESRD group, IL-8 and CRP were significantly higher in GCF, whereas IL-6 and CRP were significantly higher in serum, compared with HC group (all *P* < 0.05). In the case of GCF, taurine levels were positively correlated with IL-8 levels in both groups (all *P* < 0.05). In the case of serum, l-phenylalanine and *p*-hydroxyphenylacetic acid levels were positively correlated with CRP levels in both groups (all *P* < 0.05). Significant positive correlations were observed between metabolites (including pseudouridine, l-phenylalanine, and *p*-hydroxyphenylacetic acid) and IL-6 levels only in ESRD group.

**Conclusions:**

IL-8 and CRP are potential inflammatory makers that reflect the periodontal health of ESRD individual, which may be considered the valuable predictive diagnostics in the agreement with PPPM philosophy. Besides, metabolites of taurine in GCF as well as l-phenylalanine and *p*-hydroxyphenylacetic acid in serum are possible biomarkers correlated with inflammatory markers. All these biomarkers may also be highly recommended as a novel predictive/diagnostic tool for the assessment of inflammatory status from the perspectives of PPPM in view of susceptible population and individual screening.

## Introduction

### End-stage renal disease as the healthcare challenge

End-stage renal disease (ESRD) refers to the end stage of chronic kidney disease (CKD) that requires renal replacement therapy, including peritoneal dialysis, hemodialysis, or kidney transplantation [1]. ESRD patients with hemodialysis therapy have a relatively lower life expectancy than the general population, whose 5-year survival rate is estimated to be 76% [2]. Evidence from observational studies suggests that a larger proportion of patients undergoing dialysis treatment suffer from periodontal diseases [3]. And it is reported that 58.9% of hemodialysis patients have moderate to severe periodontitis [4]. Currently, a number of studies have shown the association between periodontal health and ESRD [4–7].

### End-stage renal disease as a risk factor of periodontal health

According to a recent systematic review, there are conflicting conclusions about the reciprocal relationship between CKD and periodontal disease, and the evidence that CKD has an impact on periodontal status is weak [7]. Immune response to the etiological factors of periodontal diseases is the determinant of disease susceptibility. ESRD patients are more susceptible to periodontal disease due to general debilitation and depression of the immunological system. The altered saliva composition and persistent low-grade inflammation in patients with CKD also mechanistically influence the onset and/or progression of periodontal disease [8, 9]. In the progression of CKD, more cytokines were secreted by inflammatory cells to the inflammation sites, which aggravates the periodontal disease [10, 11]. CKD, especially ESRD, may affect periodontal condition through systemic immune disorders, decreased saliva secretion, and accumulation of waste products in the blood [12–15].

### IL-8 as an indicator of periodontal health status

Studies based on GCF analysis exhibit the examination of the levels of inflammatory chemokines such as IL-8 can be used to assess periodontal health status [16–18]. IL-8 is an important chemokine and can be produced by many cell types in gingival epithelial tissues such as endothelial cells and neutrophils [19, 20]. Higher expression of IL-8 can be detected when the gingival tissue is in an inflammatory state in comparison to healthy gingiva [21, 22].

### The role of metabolites in pathogenesis of periodontal disease

In addition to inflammatory chemokines as the biological markers to the periodontal inflammation, the dynamics of the metabolic composition in GCF also hints the development of periodontal disease. Previous studies have reported that significant differences in metabolites related to inflammation, oxidative stress, tissue degradation, and bacterial metabolism could be observed in GCF between the population with and without periodontitis [23, 24]. Oxidative stress is a potential mediator of the progression of periodontal disease [25]. Some metabolic pathways such as purine metabolism serve as an indication of an increased inflammatory response and are associated with oxidative stress [26]. Glucose metabolism is reported to play an important role in the oral bacterial metabolism [27]. Metabolites can not only reflect metabolic activities but also affect clinical phenotypes, as they are the end products of biological processes and indicate the expression levels of many functional genes and proteins [28]. Metabolite changes in GCF could serve as a useful tool to reflect the periodontal inflammation status.

### Working hypothesis

Considering these previous observations, the levels of certain metabolites may be associated with inflammatory levels in both GCF and serum. Inflammatory markers can be an effective diagnostic tool to assess the periodontal status of individuals, especially of the ESRD population. And the change of metabolites can serve as novel predictive/diagnostic tool for periodontal health. Therefore, the aim of present study was to compare the periodontal health–related inflammatory markers and metabolite levels in serum and GCF between ESRD group and HC group using metabolomics, and to explore the association between inflammatory markers and metabolites. Integration of inflammatory and metabolomic data with clinical data will provide more valuable information for PPPM in GCF and serum of the individuals, especially of ESRD patients.

## Materials and methods

### Participants

ESRD patients undergoing hemodialysis were enrolled from Department of Nephrology, Shanghai Ninth People’s Hospital, Shanghai, China, and healthy individuals (healthy controls, HC group) were recruited from the Physical Examination Center of the same hospital from February 2019 to June 2020. The inclusion criteria for the ESRD group were age between 20 and 75 years and diagnosis of ESRD with a stable hemodialysis status. The exclusion criteria were as follows: having taken periodontal treatment within the last 6 months, antibiotic intake within 3 months, pregnancy or lactating status, presence of less than 15 natural teeth excluding third molars, presence of other systemic diseases except hypertension and diabetes (e.g., rheumatoid arthritis, systemic lupus erythematosus, psoriasis) or other severe systemic infections, and immunosuppressive therapy within last 30 days. The HC group were participants who were systemically healthy without kidney diseases. The other inclusion and exclusion criteria were the same as those for the ESRD group. All healthy controls were age-matched and sex-matched with patients undergoing hemodialysis.

Prior to the implementation of the study, a full-mouth periodontal examination was performed on all participants by an examiner using a sterile periodontal probe (PCPUNC 15, Hu-Friedy, Chicago, IL, USA). Periodontal pocket depth (PPD), gingival bleeding index (GBI), and calculus index (CI) were recorded to identify the periodontal health status. Structured questionnaire was used to collect the information on sociodemographic background (age and sex), smoking habit, oral health–related behaviors (toothbrushing frequency and use of dental floss), and medical history (presence of hypertension or diabetes) of each participant.

Ethical approval was obtained from the Research Ethics Committee of Shanghai Ninth People’s Hospital. Each participant was informed about the purpose and procedure of the study. Each participant signed a written informed consent prior to the implementation of the study.

### Collection of biological samples

#### Serum

A total of 3 ml peripheral blood was collected in vacutainer tubes in the morning from all participants. The blood samples were incubated at room temperature for not more than 2 h and centrifuged for 10 min at 2000 × *g* at 4 ℃. Serum was collected in the empty tubes and then stored at − 80 °C.

#### GCF

All participants were instructed not to eat or drink within 2 h prior to the collection of GCF. PerioPaper Strips® (Oraflow Inc., New York, USA) were used to collect GCF samples from the disto-buccal and mesio-buccal sites of all the first molars. The targeted area was separated with cotton rolls, PerioPaper Strips® were placed in the gingival sulcus for 30 s to collect GCF, and all periopaper strips from different sites were pooled in the same tube. The samples were then stored in an empty tube at − 80 °C.

### Estimation of inflammatory markers in GCF and serum

According to the kit instructions, the concentrations of IL-1β, IL-6, and IL-8 in GCF and serum as well as CRP in GCF were measured using Human High-Sensitivity T Cell Magnetic Bead Panel (Luminex Corporation, Austin, TX, USA). Furthermore, the concentration of CRP in serum was measured using a CRP ELISA kit (R&D Systems, Minneapolis, MN, USA).

### Metabolite extraction

For metabolite extraction from GCF, 200 μl extraction solution (methanol:acetonitrile:water = 2:2:1, with isotopically labeled internal standard mixture) was added to periopaper strips. The mixture was vortexed for 30 s and ground at 35 Hz for 4 min. Thereafter, the samples were sonicated for 15 min in ice-water bath. For the extraction of serum metabolites, 50 μl of sample was mixed with 200 μl of extraction solution (acetonitrile:methanol = 1:1, containing isotopically labeled internal standard mixture). The mixture was vortexed for 30 s and sonicated for 10 min in an ice-water bath. Both mixtures were incubated for 1 h at − 40 °C and centrifuged at 12,000 rpm for 15 min at 4 °C. The supernatant was separated and mixed with quality control (QC) samples for machine testing.

### Metabolomics analysis

LC/MS was used for metabolomics analysis. It was performed using a UHPLC system (Vanquish, Thermo Fisher Scientific) containing a UPLC BEH Amide column (2.1 mm × 100 mm, 1.7 μm) with Q Exactive HFX mass spectrometer (Orbitrap MS, Thermo). HPLC contained aqueous phase (25 mmol/l ammonium acetate and 25 mmol/l ammonia) and acetonitrile. The analysis was processed with elution gradient. Other LC parameters were as follows: mobile phase flow rate: 0.5 ml/min, column temperature: 30 °C, sample pan temperature: 4 °C, and injection volume: 4 μl. The QE HFX mass spectrometer was used to collect primary and secondary mass spectrometry data under the control of the acquisition software (Xcalibur, Thermo).

### Data preprocessing and annotation

The raw data was transformed into the mzXML format, using the R software to process peak detection, extraction, alignment, and integration. Furthermore, an in-house MS2 database (BiotreeDB) was used for metabolite annotation with the cutoff for annotation set at 0.3.

### Sample size

A pilot study (*n* = 15 in each group) was performed to obtain information about inflammatory markers. The levels of IL-8 were significantly different between two groups, which we chose as the primary outcome. We assumed two-sided hypothesis testing under the 5% type I error and 90% statistical power to detect a difference of 0.815 ng/ml with estimated group standard deviations of 1.331 ng/ml and 0.603 ng/ml. A total of 36 participants were required in each group, which was calculated using PASS 11.0 (NCSS, LLC*.* Kaysville, Utah, USA).

### Statistical data analysis

The Kolmogorov–Smirnov test was performed to test the normality of data distribution. Numerical variables were presented as mean (SD) when parameters were normally distributed or as median (interquartile range, IQR) when the distribution was skewed. Student’s *t*-test or Mann–Whitney *U* test was used to compare the differences between ESRD and HC groups depending on whether variances were homogenous and normal. Chi-square test or Fisher’s exact test was used to compare the differences of sociodemographic background, lifestyle, and medical history between the two groups. Based on the SIMCA software V16.0.2 (Sartorius Stedim Data Analytics AB, Umea, Sweden), principal component analysis (PCA) was used to show the sample distribution within the two groups. Orthogonal partial least-squares discriminant analysis (OPLS-DA) was also used to assess total differences of metabolites between the two groups. Multivariate models were performed to determine various metabolites with variable importance (VIP) values > 1.0 and *P* < 0.05 as the key metabolites for a further analysis. Pearson correlation coefficient was used to calculate the correlations between metabolites and inflammatory markers. Multiple factor ANOVA was used to assess the association between system diseases and the levels of IL-8 in the CGF. *P* < 0.05 was considered to be statistically significant.

## Results

### Participant characteristics

A total of 96 participants were enrolled in the study. Among these, 52 were ESRD patients receiving hemodialysis treatment (30 males and 22 females; group designated as ESRD), and 44 were healthy controls (25 males and 19 females; group designated as HC). Characteristics of all patients are summarized in Table 1. There were no significant differences regarding age, smoking history, and oral health–related behaviors between the two groups (all *P* > 0.05). The proportions of participants with diabetes and hypertension were significantly higher in the ESRD group than in the HC group (all *P* < 0.05). There were significant differences in periodontal parameters including PPD, GBI, and CI between two groups (all *P* < 0.05).

**Table 1:** Participant characteristics of the ESRD and HC groups

		ESRD group	HC group	*P* value
Total ; n		52	44	
Age groups; *n* (%)				0.574
	≤ 40	4 (7.7%)	5 (11.4%)	
	41–50	10 (19.2%)	11 (25.0%)	
	51–60	17 (32.7%)	16 (36.4%)	
	≥ 61	21 (40.4%)	12 (27.2%)	
Sex; *n* (%)				0.548
	Male	30 (57.7%)	25 (56.8%)	
	Female	22(42.3%)	19 (43.2%)	
Smoking history; *n* (%)				0.462
	Yes	15 (28.8%)	14 (31.8%)	
	No	37 (71.2%)	30 (68.2%)	
Toothbrushing frequency				0.064
	≤ 1 per day	25 (48.1%)	13 (29.5%)	
	≥ 2 per day	27 (51.9%)	31 (70.5%)	
Use of dental floss				0.222
	Yes	5 (9.6%)	8 (18.2%)	
	No	47 (90.4%)	36 (81.8%)	
Diabetes history*; *n* (%)				0.019
	Yes	13 (25.0%)	3 (7.0%)	
	No	39 (75.0%)	40 (93.0%)	
Hypertension history*; *n* (%)				< 0.001
	Yes	40 (76.9%)	10 (23.3%)	
	No	12 (23.1%)	33 (76.7%)	
GBI (%), median (IQR)		20.60 (17.86–23.68)	14.29 (12.96–17.86)	0.008
CI (%), median (IQR)		10.79 (7.41–12.00)	7.28 (3.70–10.71)	0.013
PPD (mm), median (IQR)		3.46 (3.33–3.62)	3.32 (3.19–3.44)	0.020

*ESRD*, end-stage renal disease; *HC*, healthy controls; *PPD*, periodontal pocket depth; *GBI*, gingival bleeding index; *CI*, calculus index; *IQR*, interquartile range

^*^One missing value

### Comparison of inflammatory markers in serum and GCF

The levels of IL-8 and CRP of GCF were significantly higher in the ESRD group than in the HC group (*P* = 0.032 and *P* < 0.001, respectively). In addition, the levels of IL-6 and CRP of serum were higher in the ESRD group (*P* = 0.008 and *P* < 0.001, respectively). However, there were no significant differences on the IL-1β and IL-6 levels of GCF between two groups. Moreover, IL-1β and IL-8 levels in serum did not show significant differences between the two groups (Table 2).

**Table 2:** Comparison of inflammatory markers between the ESRD and HC groups

Inflammatory markers	ESRD group Median (IQR)	HC group Median (IQR)	*P* value
Serum			
IL-1β (pg/ml)	0.75 (0.60–1.01)	0.88 (0.60–1.25)	0.225
IL-6 (pg/ml)	5.76 (4.89–7.68)	4.46 (3.36–5.03)	0.008
IL-8 (pg/ml)	46.55 (38.98–82.49)	38.86 (32.29–47.41)	0.073
CRP (ng/ml)	2.20 (1.60–4.09)	0.74 (0.50–1.26)	< 0.001
GCF			
IL-1β (ng/ml)	0.26 (0.20–0.37)	0.34 (0.20–0.53)	0.186
IL-6 (pg/ml)	2.75 (2.14–3.23)	2.46 (2.19–3.24)	0.622
IL-8 (ng/ml)	1.44 (0.81–2.26)	0.88 (0.55–1.23)	0.032
CRP (ng/ml)	0.48 (0.29–0.78)	0.16 (0.11–0.23)	< 0.001

*ESRD*, end-stage renal disease; *HC*, healthy controls; *IL-1β*, interleukin-1β; *IL-6*, interleukin-6; *IL-8*, interleukin-8; *CRP*, C-reactive protein; *IQR*, interquartile range

### Results of multiple factor ANOVA of the level of IL-8 in the GCF

Since the distributions of diabetes and hypertension history between ESRD and HC groups were significantly different, multiple factor AVOVA was performed to explore the association between groups (ESRD and HC groups), diabetes, hypertension history, and IL-8 levels in the GCF. As shown in Table 3, only the group was significantly associated with the IL-8 levels of GCF (*β* = 1.17; *P* = 0.001), while diabetes history and hypertension history were not associated with IL-8 level (*β* = 0.27, *P* = 0.486 and *β* =  − 0.61, *P* = 0.071).

**Table 3:** Results of multiple factor ANOVA analysis of the level of IL-8 in the GCF

Variables		Beta	95% CI	*P* value
Group				0.001
	ESRD	1.17	0.49–1.84	
	HC^a^			
Diabetes				0.486
	Yes	0.27	− 0.5–1.04	
	No^a^			
Hypertension				0.071
	Yes	− 0.61	− 1.28–0.05	
	No^a^			

*R*^2^ = 0.130

*GCF*, gingival crevicular fluid; *IL-8*, interleukin-8; *ESRD*, end-stage renal disease; *HC*, healthy controls; *CI*, confidence interval

^a^Reference group

### Metabolomics analysis of serum samples

**Fig. 1:**
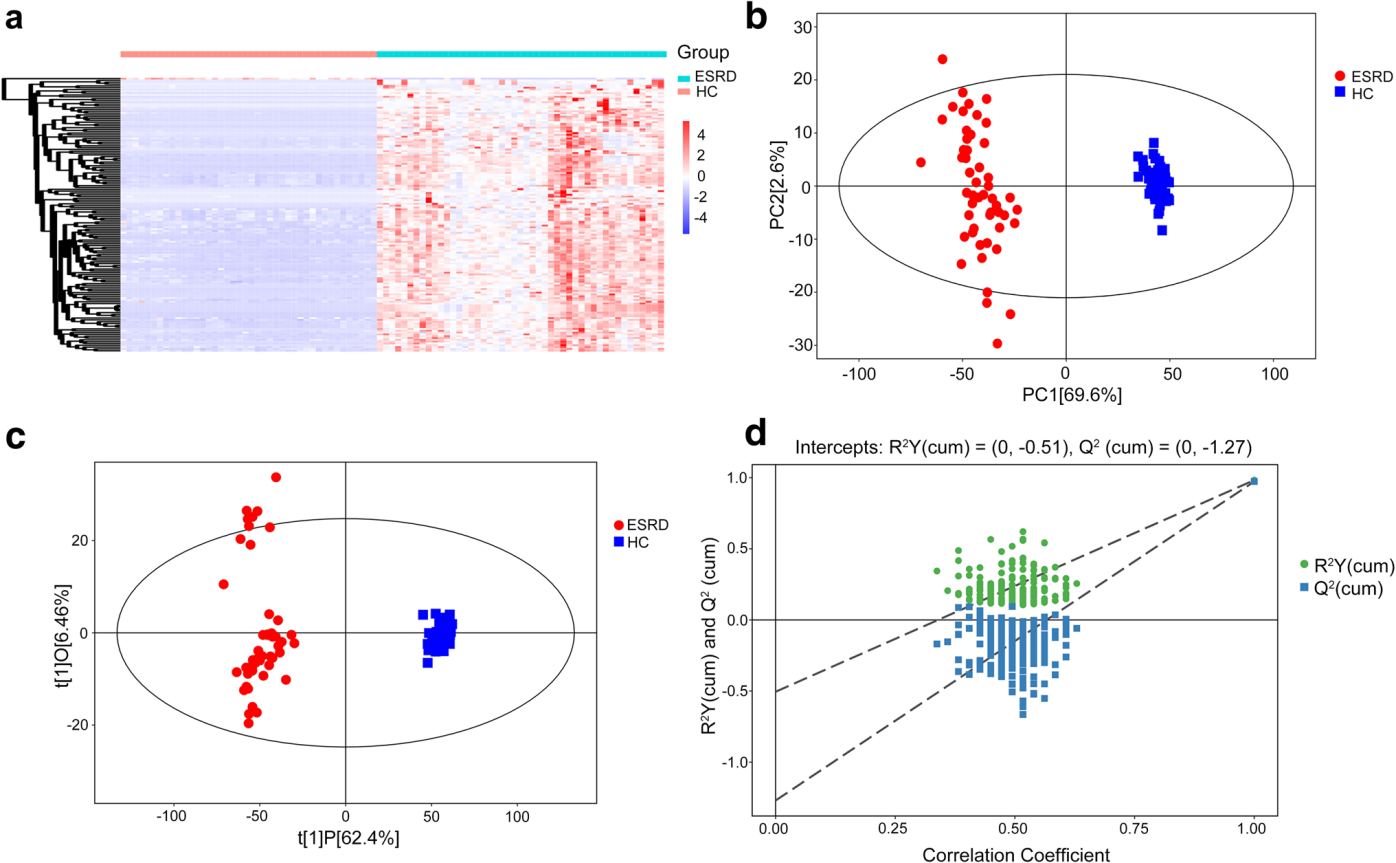
Metabolic profile of serum based on LC–MS. **a** Heatmap of metabolites between the ESRD group and the HC group. **b** PCA score plot model of serum samples. **c** OPLS-DA score plot of serum samples. **d** OPLS-DA permutation plot of serum samples. LC–MS, liquid chromatography/mass spectrometry; ESRD, end-stage renal disease; HC, healthy controls; OPLS-DA, orthogonal partial least-squares discriminant analysis; PCA, principal component analysis

A total of 261 differential metabolites with VIP > 1 and *P* < 0.05 were identified in the serum samples for the hierarchical cluster analysis (Fig. 1a). In the plot of PCA scores, the obvious divergence of metabolites in serum samples could be observed between ESRD group and HC group (Fig. 1b). The OPLS-DA model (Fig. 1c) was constructed many times to obtain the *R*^2^*Y* and *Q*^2^ values of the random model (*R*^2^*Y* = 0.983 and *Q*^2^ = 0.973), demonstrating that the original model had a good robustness and there was no overfitting (Fig. 1d). As shown in Table 4, the four main metabolic pathways that differed dramatically in serum included arginine and proline metabolism, phenylalanine metabolism, glycine/serine/threonine metabolism, and pyrimidine metabolism. Among these various pathways, 19 key metabolites were selected for further analysis, and fold changes (FCs) of key metabolites (*P* < 0.05) were calculated. The metabolites including 4-hydroxybenzoic acid, 4-acetamidobutanoic acid, and phenylacetylglutamine (log_2_ (FC): 7.287, 7.456, and 7.459, respectively) showed the highest FCs in the ESRD group in comparison with HC group. To assess the discriminating accuracy of the metabolites in differentiating two populations, the area under curve (AUC) was employed to identify 19 metabolites of serum. And the high AUCs of metabolites were observed, most of which were up to 1.00.

**Table 4:** Differential metabolites of serum between patients with ESRD and healthy controls

Metabolic Pathway	Metabolites	Mean (ESRD)	Mean (HC)	Fold change	Log_fold change	AUC value
Glycine, serine, and threonine metabolism	Glycine	1.9834	0.6171	3.214	1.684	0.972
	l-Serine	1.3163	0.1549	8.497	3.087	0.995
	l-Allothreonine	3.0007	0.4912	6.109	2.611	0.971
Phenylalanine metabolism	l-Phenylalanine	46.5813	19.7719	2.356	1.236	0.975
	*ortho*-Hydroxyphenylacetic acid	15.8486	0.3832	41.359	5.370	1.000
	Phenylacetic acid	15.8486	0.3832	41.359	5.370	0.997
	Phenylacetylglutamine	174.1466	0.9897	175.951	7.459	1.000
	4-Hydroxybenzoic acid	13.9005	0.0890	156.181	7.287	1.000
	*p*-Hydroxyphenylacetic acid	2.1442	0.0570	37.632	5.234	0.993
Pyrimidine metabolism	Cytosine	2.0971	0.1987	10.556	3.400	1.000
	Deoxyribose 1-phosphate	542.9885	12.8789	42.161	5.398	0.983
	Uracil	6.0958	1.5430	3.951	1.982	0.995
	Pseudouridine	34.0287	1.6156	21.063	4.397	1.000
Arginine and proline metabolism	Creatinine	75.2642	2.7376	27.493	4.781	1.000
	Citrulline	4.3835	0.0979	44.772	5.485	1.000
	l-Proline	38.7071	12.2175	3.168	1.664	0.975
	4-Hydroxyproline	4.1395	0.5685	7.281	2.864	0.970
	4-Acetamidobutanoic acid	10.1351	0.0577	175.635	7.456	1.000
	*N*-Acetylglutamic acid	0.9101	0.0517	17.611	4.138	1.000

*ESRD*, end-stage renal disease; *HC*, healthy controls; *AUC*, area under curve

### Metabolomics analysis of GCF samples

**Fig. 2:**
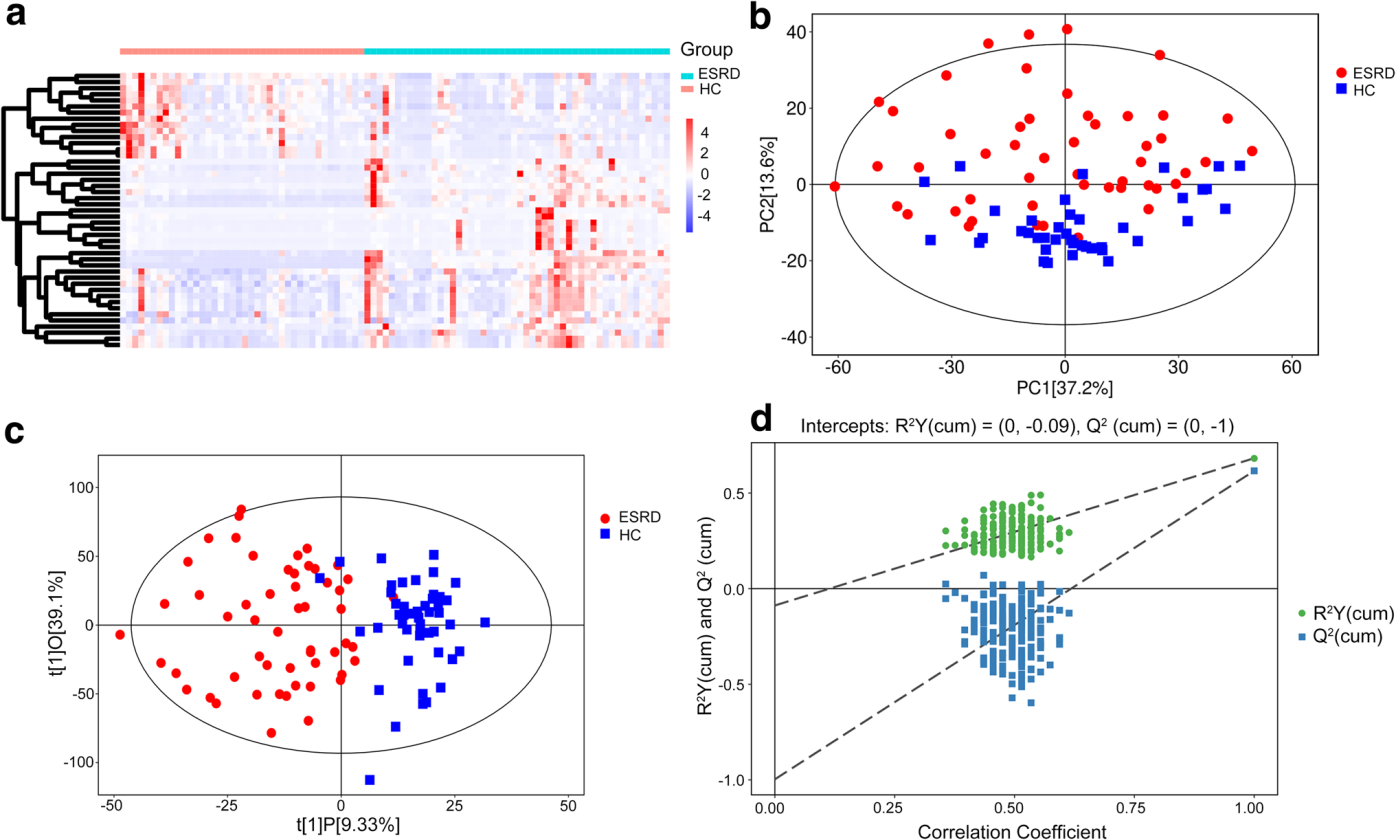
Metabolic profile of GCF based on LC–MS. **a** Heatmap of metabolites between ESRD group and HC group. **b** PCA score plot model of GCF samples. **c** OPLS-DA score plot of GCF samples. **d** OPLS-DA permutation plot of GCF samples. LC–MS, liquid chromatography/mass spectrometry; ESRD, end-stage renal disease; HC, healthy controls; OPLS-DA, orthogonal partial least-squares discriminant analysis; PCA, principal component analysis

A total of 88 differential metabolites with VIP > 1 and *P* < 0.05 were identified for the hierarchical cluster analysis (Fig. 2a). The PCA score showed a good separation of metabolites in GCF between the two groups (Fig. 2b). The OPLS-DA model (Fig. 2c) was constructed many times to obtain the *R*^2^*Y* and *Q*^2^ values of the random model (*R*^2^*Y* = 0.682 and *Q*^2^ = 0.616, Fig. 2d). As shown in Table 5, the seven main metabolic pathways that showed the significant differences in GCF samples were galactose metabolism, nicotinate and nicotinamide metabolism, phenylalanine metabolism, purine metabolism, starch and sucrose metabolism, taurine and hypotaurine metabolism, and pyrimidine metabolism based on the KEGG pathway enrichment analysis (*P* < 0.05). The metabolites significantly upregulated in ESRD group in comparison to HC group were raffinose, alpha-d-glucose, d-maltose, dUMP, and N1-methyl-2-pyridone-5-carboxamide. Other compounds, including taurine, 3-sulfinoalanine, xanthine, inosine, hypoxanthine, *m*-coumaric acid, hydrocinnamic acid, uridine, and niacinamide, were downregulated in the ESRD group. Metabolites with the greatest FCs (*P* < 0.05) that were increased in ESRD group relative to HC group were dUMP, alpha-d-glucose, and raffinose (log_2_ (FC): 3.120, 3.164, and 5.017, respectively). Two metabolites—niacinamide and hydrocinnamic acid—with the greatest FCs were decreased (*P* < 0.05) in the ESRD group compared to the HC group (log_2_ (FC): − 1.479 and − 1.708, respectively). The AUCs of 14 metabolites in the GCF can be observed in Table 5, and almost all metabolites were above 0.700.

**Table 5:** Differential metabolites of GCF between patients with ESRD and healthy controls

Metabolic pathway	Metabolites	Mean (ESRD)	Mean (HC)	Fold change	Log_fold change	AUC value
Nicotinate and nicotinamide metabolism	Niacinamide	0.0361	0.1008	0.359	− 1.479	0.703
	*N*1-Methyl-2-pyridone-5-carboxamide	0.3889	0.0505	7.701	2.945	0.952
Phenylalanine metabolism	*m*-Coumaric acid	1.5604	2.4824	0.629	− 0.670	0.748
	Hydrocinnamic acid	10.0707	32.8998	0.306	− 1.708	0.823
Taurine and hypotaurine metabolism	Taurine	5.7182	9.2706	0.617	− 0.697	0.778
	3-Sulfinoalanine	1.1994	2.0963	0.572	− 0.806	0.747
Purine metabolism	Inosine	0.1574	0.3363	0.468	− 1.095	0.843
	Xanthine	0.9294	1.2673	0.733	− 0.447	0.687
	Hypoxanthine	26.5315	42.3163	0.627	− 0.674	0.768
Galactose metabolism	alpha-d-Glucose	0.0889	0.0099	8.961	3.164	0.714
	Raffinose	0.0408	0.0013	32.389	5.017	0.740
Starch and sucrose metabolism	d-Maltose	0.5640	0.0761	7.413	2.890	0.744
Pyrimidine metabolism	Uridine	2.9903	5.2445	0.570	− 0.810	0.793
	dUMP	0.0358	0.0041	8.692	3.120	0.709

*GCF*, gingival crevicular fluid; *ESRD*, end-stage renal disease; *HC*, healthy controls; *AUC*, area under curve

### Correlation between inflammatory markers and key metabolites in serum and GCF

The correlations between key metabolites found in serum and GCF and four inflammatory markers including IL-1β, IL-6, IL-8, and CRP were calculated. Only statistically significant positive or negative correlations (*P* < 0.05) between the two groups are shown in Table 6. In the serum samples, the levels of l-phenylalanine and *p*-hydroxyphenylacetic acid were positively correlated with the levels of CRP in both groups but were positively correlated with the levels of IL-6 only in the ESRD group. Significant positive correlation was observed between pseudouridine and IL-6 only in ESRD group. In the case of GCF samples, the levels of taurine were positively correlated with IL-8 levels in the ESRD group and HC group, whereas 3-sulfinoalanine levels were positively correlated with IL-8 levels only in the ESRD group.

**Table 6:** Correlation between various inflammatory markers and key metabolites in serum and GCF between the two groups

	ESRD group	HC group
*p*-Hydroxyphenylacetic acid and IL-6	0.519^**^	− 0.081
l-Phenylalanine and IL-6	0.492^**^	− 0.146
Pseudouridine and IL-6	0.377^**^	− 0.132
*p*-Hydroxyphenylacetic acid and CRP	0.324^*^	0.309^*^
l-Phenylalanine and CRP	0.309^*^	0.507^**^
Taurine and IL-8	0.570^**^	0.416^**^
3-Sulfinoalanine and IL-8	0.469^**^	0.016

*ESRD*, end-stage renal disease; *HC*, healthy controls; *GCF*, gingival crevicular fluid; *IL-6*, interleukin-6; *IL-8*, interleukin-8; *CRP*, C-reactive protein

^*^P < 0.05

^**^P < 0.01

## Discussion

### Achievements in the present study

The significant associations between periodontal health status and ESRD have been investigated among a number of studies [4–7, 29]. It is evident that patients with kidney diseases have poor periodontal health in comparison to healthy individuals [30, 31]. However, no study could be traced about comparing GCF metabolites between ESRD patients and the healthy as well as the association between metabolites and periodontal inflammation status; thus, the present study was conducted to compare inflammation markers, the significantly altered metabolites, and their potential associations between two groups. In the present study, we found that IL-8 and CRP levels of GCF in ESRD group were significantly higher than those in the HC group, in which IL-8 was positively correlated with taurine and 3-sulfinoalanine. Meanwhile, we observed the periodontal parameters (PPD, GBI, and CI) of ESRD patients were worse than that of healthy participants.

### Result interpretation of IL-8 in the pathogenesis of periodontal disease

According to the 2018 American Academy of Periodontology and European Federation of Periodontology, periodontal health would be defined as a clinically non-inflammatory status, which means that absence of inflammation in periodontal tissue is a prerequisite for defining periodontal health [32]. And the success of treatment for periodontitis is to minimize inflammation and improve periodontal parameters [32]. We utilized inflammatory markers, especially IL-8, to reflect the periodontal inflammation status between the groups with and without ESRD. IL-8 can help neutrophils migrate from gingival tissue to the gingival crevice, thus facilitates the inflammatory cell infiltration and release of granule enzymes to efficiently degrade periodontal connective tissue [20, 33]. Previous studies proved that IL-8 was mainly detected in deeper layers of the pocket epithelium and indicated that IL-8 was involved in the induction and development of periodontal disease [21, 34, 35]. Dag A et al. reported that IL-8 levels in GCF were found higher in hemodialysis patients compared with the healthy individuals [36]. The finding was consistent with our study. IL-8 may play an important role in the periodontal condition of ESRD patients.

### Metabolite as a tool for predicting periodontal inflammation status

When periodontal tissue is in an inflammatory state, inflammatory factors such as cytokines, bacterial antigens, various cells, metabolites, and other degradation products are released in the GCF [37]. Interestingly, IL-8 showed a significant positive correlation with taurine and 3-sulfinoalanine in our study. Taurine, a type of free amino acid with antimicrobial and antioxidant activities, has a great impact on important biological processes related to inflammation [38, 39]. It was reported that taurine could inhibit oxidation of hypochloric acid associated with myeloperoxidase-hydrogen peroxide-halide system of neutrophils [38]. 3-Sulfinoalanine, a product of cysteine dioxygenase, is involved in several processes, such as pyruvate production and taurine/hypotaurine synthesis [40]. The finding suggests that abnormally elevated inflammatory markers may induce the production of taurine and 3-sulfinoalanine to inhibit the inflammation. We also found that other significant metabolites of GCF were more abundant in hemodialysis patients than in the healthy controls. However, the mechanism is not clear due to a shortage of the relevant researches.

### Discrepancies between serum and GCF with regard to metabolites and inflammatory markers

Furthermore, though the compositions of GCF mainly originated from serum [37], there were many discrepancies between serum and GCF with regard to metabolites and inflammatory markers. IL-6 levels were significantly different in the serum between two groups rather than IL-8 levels. IL-6-associated and CRP-associated metabolites including pseudouridine, l-phenylalanine, and *p*-hydroxyphenylacetic acid were reported to be associated with renal disease [41–43], which were seen obviously abundant in the serum instead of GCF in the ESRD patients. The discrepancies between serum and GCF exist due to the interaction of host defense, oral microbial species, and external factors [37]. Evidence supports ESRD could make periodontal microbial community different from that of those without ESRD [44]. The interplay between microbial agent and the adjacent periodontal tissues often increases gingival inflammation and releases biologically active substances [45].

### PPPM concept in the current study

Early prediction and prevention of diseases using precise monitoring methods is a key paradigm in healthcare [46]. GCF, a kind of non-invasive tissue fluid and easily processed, contains tremendous metabolites from the host as well as bacteria [47, 48]. GCF is likely to reflect the periodontal health status [48]. ESRD patients are susceptible to infection due to the impairment of immune system, leading to an increase of proinflammatory cytokines [11]. Our study indicated that there were some correlations between key metabolites and inflammation. Therefore, the inflammatory markers and metabolites in GCF may be valuable biomarkers for patient stratification, disease surveillance, predictive diagnosis, and targeted prevention. Through the omics analysis of GCF, we can determine unique metabolite information and then analyze the related molecular pathways in global terms, which can predict whether there is any disorder in periodontal tissue or system health [49]. Specifically, based on the results in this study, the individuals may be in a state of periodontal inflammation and at a risk of periodontal disease when the most direct biomarkers such as taurine in GCF decreased greatly compared with the previous levels or were at a low level. The prediction can trigger targeted preventions, such as increased attention to oral hygiene (e.g., the frequency of tooth brushing) and regular oral care (e.g., supragingival scaling) [50]. Meanwhile, the metabolites with anti-inflammatory and antioxidant properties found in this study can aid in understanding the pathogenesis of periodontal disease of ESRD patients and help in the development of therapeutics.

### Strengths and limitations

Some limitations of this study need to be addressed. Selection bias may exist as the proportions of participants with the history of diabetes and hypertension were uneven in the two groups; The bias is difficult to avoid as diabete**s** and hypertension are the leading causes for ESRD, account for 61% of dialysis cases [51–53]. However, multiple factor analysis was performed to analyze the associations between the inflammatory marker and these two variables. And the result showed hypertension and diabetes were not significantly associated with the IL-8 levels of GCF. Secondly, clinical attachment loss was not included in periodontal parameters of the study as it was challenging and time-consuming to measure gingival recession and cemento-enamel junction in the chairside operation [54]. Much work has already been done to focus on the interaction between ESRD and periodontal health. However, the relationship between periodontal and kidney diseases has not been explored deeply so far. Our results provide some clues regarding their relationships based on significant differences of inflammatory markers and metabolites. To the best of our knowledge, it is the first study that reported the metabolites and its association with inflammation in the GCF of ESRD patients, the extent to discuss these findings is limited for us. The role of the hemodialysis may lead to the poor periodontal health status [6]. However, further evidence is certainly needed to draw causal conclusions in the future.

### What is known about liquid biopsy in COVID-19 management?

The novel Coronavirus Disease 2019 (COVID-19) is the most dramatic healthcare crisis that has triggered a significant shock in the worldwide economy with quarantine policies [55]. It was reported that CKD seemed to be associated with enhanced risk of severe COVID-19 infection [56]. During the COVID-19 pandemic, it is a great challenge for the management of CKD patients needing dialysis treatment at the hospital as they cannot respect the quarantine, and there is no guarantee that they correctly follow the preventive measures at home [57]. Furthermore, they may ignore oral health care and other potential problems due to the length of time spent in the dialysis center, leading to the delay of diagnosis and treatment of some oral diseases like periodontitis [36]. Patients with CKD should hence be advised to take extra non-invasive and simple methods to minimize risk of the exposure to other resource infection. As a non-invasive diagnostic method, liquid biopsy can obtain lots of information about disease from liquid samples, such as GCF. Detection of inflammatory and metabolic markers is highly indicative for periodontal health status of individuals, especially for those ESRD patients with compromised immunity.

## Conclusions

The periodontal health status of ESRD patients appears to be worse than the average individuals. Inflammation markers including IL-8 and CRP were significantly higher in the GCF of ESRD patients, which can be indicators of periodontal health status and may be considered the valuable predictive diagnostics especially in the ESRD patients, who are susceptible to the infection due to systemic immune disorders. Besides, metabolites of taurine in GCF as well as l-phenylalanine and *p*-hydroxyphenylacetic acid in serum are possible biomarkers correlated with inflammatory cytokine. All these biomarkers may also be highly recommended as a novel predictive/diagnostic tool for the assessment of inflammation status from the perspectives of PPPM in view of susceptible population and individual screening. And some metabolites such as taurine with anti-inflammatory and antioxidant properties can be applied as the targeted medicine to the patient in focus in the agreement with PPPM philosophy. Integration of inflammatory and metabolic markers in routine medical checks as a pre-health screening and judgement of disease progression will create opportunity for risk assessment, disease stratification, individualized management, and targeted preventive measures, which are all key concepts in PPPM.

## Data Availability

The datasets used or analyzed during the current study are available from the corresponding author on reasonable request.

## Abbreviations

PPPM: Predictive, preventive, and personalized medicine
GCF: Gingival crevicular fluid
ESRD: End-stage renal disease
HCs: Healthy controls
IL: Interleukin
CRP: C-Reactive protein
PDD: Periodontal pocket depth
GBI: Gingival bleeding index
CI: Calculus index
LC/MS: Liquid chromatography/mass spectrometry
IQR: Interquartile range
PCA: Principal component analysis
OPLS-DA: Orthogonal partial least-squares discriminant analysis
FC: Fold change
AUC: Area under the curve
COVID-19: Coronavirus disease 2019

## Code availability

All software applications used are included in this article.
